# Inadequate thermal refuge constrains landscape habitability for a grassland bird species

**DOI:** 10.7717/peerj.3709

**Published:** 2017-08-18

**Authors:** John M. Tomecek, Brian L. Pierce, Kelly S. Reyna, Markus J. Peterson

**Affiliations:** 1 Department of Wildlife and Fisheries Sciences, Texas A&M University, College Station, TX, USA; 2 Texas A&M Natural Resources Institute, Texas A&M University, College Station, TX, USA; 3 Department of Biological Sciences, University of North Texas, Denton, TX, USA; 4 Department of Biological Sciences, University of Texas at El Paso, El Paso, TX, USA

**Keywords:** Embryonic stress, Grassland birds, *Colinus virginianus*, Northern bobwhite, Quail, Microclimate, Land use change, Thermal refuge, Grassland ecosystem

## Abstract

Ecologists have long recognized the influence that environmental conditions have on abundance and range extent of animal species. We used the northern bobwhite (*Colinus virginianus*; hereafter bobwhite) as a model species for studying how microclimates serve as refuge against severe weather conditions. This species serves as an indicator or umbrella species for other sensitive ground-nesting, grassland obligate species. We conducted a mensurative field experiment in the rolling plains of Texas, USA, a semi-arid ecosystem on the southwestern periphery of bobwhite range, to determine whether native bunch grasses, apparently suitable for bobwhite nesting, could reduce ambient temperature below levels harmful for eggs. During the nesting season, we compared temperature and relative humidity readings at daily heat maxima (i.e., the 3 h during each day with highest temperatures) during the nesting season over the course of two years at 63 suitable nest sites paired with 63 random locations (*n* = 126) using two sensors at ∼10 and ∼60 cm above ground level. Mean temperature at nest height was 2.3% cooler at nest sites (35.99 °C ± 0.07 SE) compared to random locations (36.81 °C ± 0.07 SE); at ambient height, nest sites were slightly cooler (32.78 °C ± 0.06 SE) than random location (32.99 °C ± 0.06 SE). Mean relative humidity at nest sites was greater at nest height (34.53% ± 0.112 SE) and ambient height (36.22% ± 0.10 SE) compared to random locations at nest (33.35% ± 0.12 SE) and ambient height (35.75% ± 0.10 SE). Based on these results, cover at sites that appear visually suitable for nesting by bobwhites and other ground nesting birds provided adequate thermal refuge in the rolling plains by maintaining cooler, moister microclimates than surrounding non-nesting locations. Post-hoc analyses of data revealed that habitat conditions surrounding suitable nest sites strongly influenced thermal suitability of the substrate. Given that eggs of bobwhites and probably other species would experience lethal temperatures without these thermal refuges in the context of proper habitat condition, nesting vegetation is a critical component of niche space for bobwhites and other ground nesting birds in semi-arid regions. Many contemporary land uses, however, degrade or destroy bunch grasses and grassland systems, and thus decrease landscape inhabitability. Conservationists working with obligate grassland species that require bunch grasses in semi-arid regions should develop land management strategies that maximize the availability of these thermal refuges across space and time.

## Introduction

Ecologists have long recognized that environmental conditions influence abundance and range extent of animal species. Worldwide, extreme temperature (hot or cold) or precipitation (wet or dry periods) have long been known to influence population dynamics of diverse animal species (Birch, 1957). In arid and semi-arid regions, heat often constrains the inhabitable area of a landscape generally suited for a particular species (Cameron, 1981; Carey, 2009; Hansen, 2009; Parker & Gillingham, 1990; Salzman, 1982), driving animals to seek refuge in cooler and moister microclimates (e.g., caves, dense vegetation) (Dawson et al., 2000; Sheldon et al., 2010; Sinervo et al., 2010). In essence, habitat ceases to exist when thermal environments are unsuitable.

The northern bobwhite (*Colinus virginianus*; hereafter bobwhite), a widely distributed new world quail, is an excellent model for studying microclimate refuges against severe weather conditions because bobwhites are one of the most studied avian species, exhibit population responses to weather patterns (Bridges et al., 2001), and may act as an indicator or umbrella species for other sensitive ground-nesting or grassland obligate species (Hovick et al., 2014). The abundance and range of bobwhite are strongly influenced by various aspects of weather. It has long been recognized that extreme cold (Robinson & Baker, 1955), prolonged ice and snow coverage (Klimstra & Roseberry, 1975; Roseberry, 1964), flooding (Lehmann, 1984; Stoddard, 1931), and extreme heat (Forrester et al., 1998; Guthery et al., 2005; Johnson & Guthery, 1988) limit bobwhite production and survival. In semi-arid ecosystems on the western edge of the bobwhite’s range (Hernández & Peterson, 2007), extreme heat can render landscapes inhospitable, cause asynchronous incubation, reduce adult survival, and stunt or kill embryos, thereby limiting bobwhite production (Guthery, Land & Hall, 2001; Reyna, 2010; Reyna & Burggren, 2012).

Bobwhites nest in vegetation (i.e., native bunch grasses) that functions in maintaining cooler microclimates through the interaction of sun-shading and evaporative cooling in semi-arid landscapes (Guthery et al., 2005; Hernández & Peterson, 2007; Johnson & Guthery, 1988; Rader et al., 2007). As a result, these vegetative structures are essential components of bobwhite habitat in these regions. Drought and livestock grazing, however, often limit the availability of such vegetation in semi-arid regions, thereby reducing bobwhite production (Bridges et al., 2001; Guthery et al., 2002). Thus, lack of thermal refuges due to drought, heavy livestock grazing, or other land uses that consume or degrade essential vegetation, reduces available habitat and lowers carrying capacity below levels required for viable bobwhite subpopulations (Forrester et al., 1998; Guthery, 1999; Guthery, Peterson & George, 2000; Peterson, 2007). We suggest that the greatest contribution of nesting cover as thermal refuge for bobwhite eggs is during temperature maxima at the peak of bobwhite nesting, when availability of thermally suitable bobwhite nesting habitat is most tightly constrained.

Thermal refuges are critical for bobwhites in semi-arid ecosystems. Broad-scale weather phenomena (e.g., precipitation, temperature) influence the production of nesting vegetation that explains a large proportion of the variability in bobwhite abundance among years (Bridges et al., 2001; Guthery et al., 2002; Lehmann, 1984; Lusk et al., 2002). Although such inferences are valuable, these data were collected at a scale too broad to capture the influence of weather relevant to individual bobwhites. Severe heat loads have physiological effects on individual bobwhites at various life history stages; however, heat loads experienced by bobwhites in situ have not been adequately addressed, particularly during nesting death (Dabbert, Lochmiller & Teeter, 1997; Reyna, 2010; Reyna & Burggren, 2012). A few field studies attempted to quantify heat experienced by wild bobwhites, but were limited by small spatial and temporal extent, and did not address nesting structures (Forrester et al., 1998; Guthery, Land & Hall, 2001; Guthery et al., 2005).

We conducted a mensurative field experiment (Hurlbert, 1984) in the rolling plains of Texas (Gould, 1969), a semi-arid ecosystem on the western edge of the range of the bobwhite, to test the hypothesis that native bunch grass vegetation offers suitable habitat for bobwhite nesting by functioning as thermal refuge for bobwhites. Specifically, we monitored temperature and relative humidity within vegetation suitable for bobwhite nesting and at random points to determine whether temperature and relative humidity differed at daily heat maxima during bobwhite nesting season (roughly 1 May–31 September per Hernández & Peterson, 2007) at (1) two heights related to nest height (∼10 cm) and ambient temperature (∼60 cm) and (2) two locations related to vegetation suitable for bobwhite nesting, and paired, random points of random cover types. We discuss our results with respect to the limits of bobwhite nesting cover as thermal refuge, and extend the implications to other species that use similar microclimates to avoid harmful heat stress. We close by discussing the effects of weather-related stress on species, particularly those experiencing rapid changes in habitat availability due to land use change, and changing climatic conditions.

## Methods

### Study area

The rolling plains of Texas, USA (Gould, 1969) is a semi-arid physiographic region in northwestern Texas that historically supported large bobwhite subpopulations that are now declining in abundance (Peterson, Wu & Rho, 2002). Topography is characterized by flat to gently rolling plains intersected by streams that flow in an east to southeasterly direction; elevation ranges from 215 to 950 m (Gould, 1969). Soils vary from coarse sands to tightly packed clays, with substantial fine scale variability in soil type. Annual precipitation increases in an easterly direction, from roughly 550 mm to nearly 760 mm; May and September are the wettest months, with a summer dry period. Plant communities were dominated historically by mid or tall bunch grass species, including little bluestem (*Schizachyrium scoparium*), big bluestem (*Andropogon gerardii*), sand bluestem (*Andropogon hallii)*, Indian grass (*Sorghastrum nutans*), and sideoats grama (*Bouteloua curtipendula*). Riparian areas supported woody plant communities that included oaks (*Quercus* spp.), eastern cottonwood (*Populus delitoides*), elms (*Ulmus* spp.), and junipers (*Juniperus* spp.). Extensive woody plant encroachment due to overgrazing and/or fire suppression has resulted in reduction of grass-dominated range sites. Honey mesquite (*Prosopis glandulosa*) is a common woody species throughout the rolling plains, with shinnery oak (*Quercus havardii*) and sand sage (*Artemisia filifolia*) primarily on sandy soils. As a result, rangeland comprised of mixed native grass-herbaceous vegetation with interspersed shrubs characterizes much of the land cover. Although livestock grazing operations dominate land use across this region as a whole, many areas have been converted to cropland (USDA-NASS, 2007).

We conducted our study on six ranches dispersed across the rolling plains, which range in size from roughly 1,600 to 78,200 ha. These ranches capture much of the landscape variability (i.e., geology, soil type, vegetation, and topography) experienced by bobwhites in the rolling plains ecoregion. All six ranches were managed for a combination of livestock grazing and fee hunting. All ranches experienced drier conditions during our study than normal due to an ongoing drought. Three sites experienced wildfires during 2011 that removed all vegetation on ≥60% of those sites.

### Data collection

**Figure 1:**
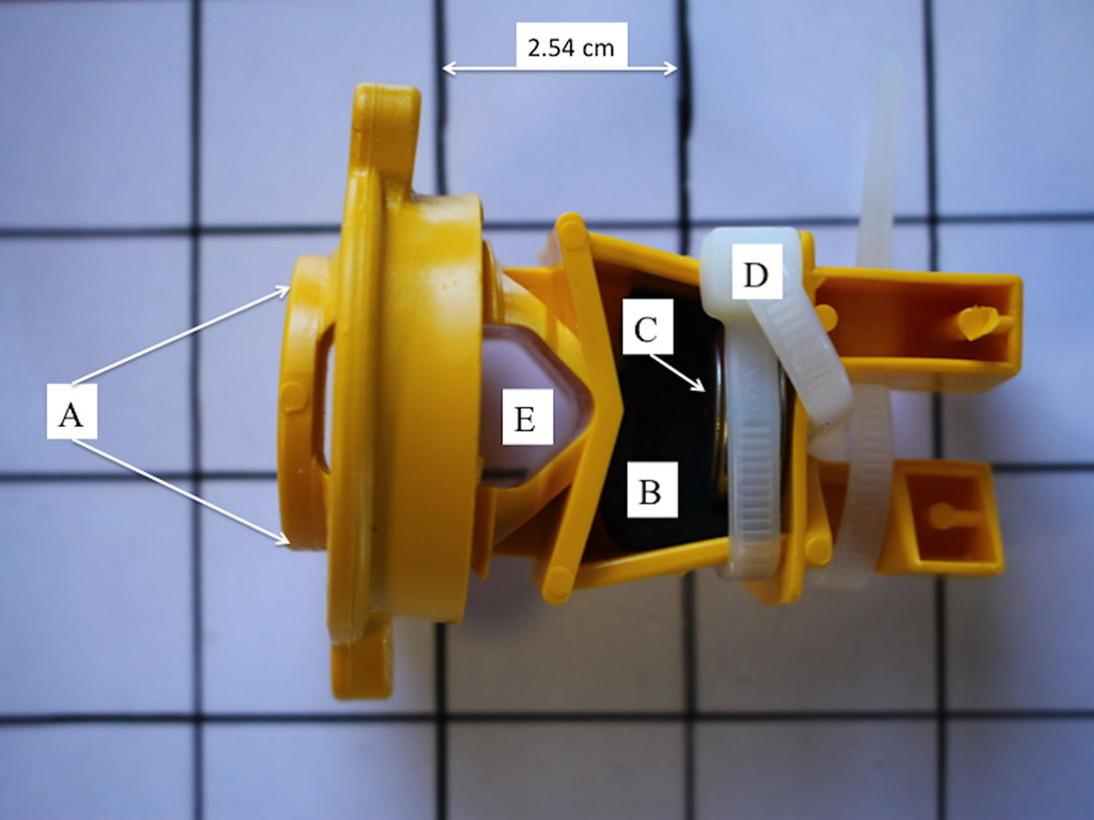
Underside view of construction and installation of mounts for ibutton temperature and relative humidity sensors. Diagram illustrating construction and insulation of mounted iButtons when viewed from the underside, specifically the (A) insulator mount, (B) foam pad, (C) iButton sensor, (D) white cable tie, and (E) attachment orifice to rebar electric fence post.

We collected temperature and relative humidity data for two years (January 2012–2014) using a spatially nested (multi-scalar) sampling design along sampling transects. On each ranch, we established sampling points with an array at each point, and a minimum separation of 1 km between points. We established nine and 10 sample arrays on two ranches because of transect length constraints, and 11 sample arrays on the other four ranches (*n* = 63). Thus, transects were nine, 10, and 11 km in length, respectively. We used model DS1923 Hygrochron iButton temperature/humidity sensors (hereafter sensors) (Dallas Semiconductor, 2011) mounted inside the open cavity on the underside of screw-on type electric fence post insulators (Fig. 1) on a 1 m piece of steel rebar. The fence post insulators protected sensors against direct insolation, weather (e.g., hail, flood), and animal damage. Mounted sensors were aligned toward the west. Four sensors at each sample array collected hourly temperature and relative humidity data at two heights (10 and 60 cm) in two locations (random point and apparently suitable cover). The 10 cm height corresponded with approximate the height of a bobwhite nest, and a height of 60 cm was used as an estimate of ambient temperature. For the purposes of this study, we defined apparently suitable nesting cover as the center of the clump of native, warm-season bunch grass suitable for bobwhite nesting nearest to the matching random point (Rader et al., 2007). Although we recognize the ability of other structures (e.g., bunch grass–brush complexes) to serve as bobwhite nesting cover, we used a structure that is ubiquitous for bobwhite nesting across the species distribution. We moved nesting sensors before each nesting season if senescence and degradation of the previous year’s nesting cover had occurred. Random points were placed 25 m from the sampling transect every 1 km of transect travelled, alternating left or right. Points were checked to ensure they were no closer together than every 1 km. These transects representatively traversed every vegetation cover type on each ranch in proportion to their abundance. Although assigned randomly, these random points were generally located on bare soils; only 3% occurred in vegetation (i.e., woody plants, grasses) that may have provided some effect on in situ temperature and relative humidity. We collected data on relative habitat quality surrounding sensor locations as it related to ability of bobwhites to use a landscape. We used ocular estimation to assess percent cover of bare ground, herbaceous cover, woody vegetation, and available nesting structures. We ranked sites from 1 to 5, indicating habitat quality that ranged from poor (1) to excellent (5) based on accepted standards attested for the rolling plains of Texas and Oklahoma in the literature (Guthery et al., 2001; Jackson, 1969; Webb & Guthery, 1982). Sensors collected temperature and relative humidity hourly for two years (January 2012–2014). We downloaded data from sensors every four months using the Thermodata Viewer Software (Thermodata Corporation, Whitewater, WI, USA). During download, we removed sensors from mounts, inspected mounts for damage, replaced damaged mounts, and replaced foam inserts and cable ties. Raw data were later reformatted, and imported into a relational database.

### Analyses

We compared temperature and relative humidity between cover types and sensor heights using repeated-measures, split-block analyses of variance (ANOVA) with one within-factor (time as month) and two between-factors (sensor height and sensor location). This method accounts for lack of independence when repeated observations are obtained from the same experimental units (Tzilkowski & Storm, 1993; von Ende, 2001; Zar, 2010). Environmental heterogeneity among ranches was controlled by blocking (Gotelli & Ellison, 2012; Zar, 2010). We examined differences in temperature and relative humidity during daily heat maxima (i.e., 1300–1500 h) in bobwhite nesting season (1 May–31 September) to compare potential for harmful heat exposure: (1) between random and nesting locations, (2) between ambient and nesting heights, and (3) across months. Critical assumptions were assessed prior to hypothesis testing (see next paragraph). All analyses were conducted in JMP Pro 11.0.0 (SAS Institute, Cary, NC, USA), and we considered *p* < 0.05 significant.

Plots of normality and residual variance indicated that temperature and relative humidity data were approximately normally distributed and homoscedastic. Mauchley’s test for sphericity (circularity; Mauchly, 1940) revealed a lack of sphericity in the temperature variance–covariance matrix (*W* = 0.38; *Χ*^2^ = 27,697.11, *p* < 0.001) and in the relative humidity variance–covariance matrix (*W* = 0.70; *Χ*^2^ = 10142.52, *p* < 0.001). We therefore used the more conservative Greenhouse–Geisser corrected *F*-test (Greenhouse & Geisser, 1959; Huynh & Feldt, 1970) to assess treatment effects for temperature (ε = 0.38) and relative humidity (ε = 0.70) data. We visually evaluated trends between treatment factors and habitat condition using both plots of mean temperature and relative humidity during daily heat maxima to assess the ability of nesting cover to reduce in situ heat below harmful levels (i.e., temperatures ≥40 °C).

Due to obvious differences in habitat condition within and across ranches due to differences in land use, precipitation, and other factors, we conducted an analysis to investigate the influence of habitat condition on thermal suitability of nesting substrates. We ranked relative habitat conditions for bobwhites at each sampling point on a scale from 1 to 5 based on ocular estimates of grass and forb availability, total percent ground cover, and diversity of vegetation from photo points taken each May and September. We conducted a one-way ANOVA to test for differences in temperature and relative humidity by habitat condition. A post-hoc test using a pairwise Student’s *t-*test comparison between all ranked habitat classes to assess whether differences in temperature in situ at all sensors were significant between different assessed habitat conditions.

## Results

### Temperature

When examining the effect of between-subjects factors (location, height) on temperature by month, we found a significant main effect between both locations and heights (Table S1). There also was a significant interaction between location and height, which indicates that monthly temperatures at both nesting and random locations were strongly influenced by height of sensor, rather than placement in suitable nesting cover or not. When considering the within-subjects factor (time) on in situ temperatures, we found a significant main effect of month (Table S1). Nevertheless, the interactions of month and location, month and height, and month, location, and height were significant, indicating that time, alone, was not the driving influence on temperature.

Mean temperature was higher at nest height in random locations ($\bar x = 36.81$, SE = 0.0683) than at nest height in nesting cover locations ($\bar x = 35.99$, SE = 0.07). Mean temperatures at ambient height in nesting and random locations were essentially identical ($\bar x = 32.78$, SE = 0.06, and $\bar x = 32.99$, SE = 0.06, respectively). During the course of our study, 54.2% of temperature readings occurred between 0 and 40 °C ($\bar x = 34.65$, SE = 0.03), a temperature range suitable for bobwhites.

### Relative humidity

For between-subjects factors influencing mean relative humidity by month, we found a significant main effect between heights, but not locations (Table S2). The presence of a significant interaction between location and height indicated that differences between locations depended primarily on height. Although we found a significant main effort for the within-subjects factor (month) on relative humidity (Table S2), interactions among month and location, month and height, and month, height, and location were significant, indicating that time was not the driving force in mean relative humidity. Thus, while the influence of time was statistically significant, in situ relative humidity is largely dependent on height, and to a lesser extent, location.

Overall, mean relative humidity was higher at nest height in nesting cover locations ($\bar x = 34.53$, SE = 0.11) than at nest height in random locations ($\bar x = 33.35$, SE = 0.12). Similarly, ambient height in nesting locations exhibit higher mean relative humidity ($\bar x = 36.22$, SE = 0.10) than ambient height in random locations ($\bar x = 35.75$, SE = 0.10). During the course of our study, 80.0% of relative humidity readings occurred between 19% and 100% relative humidity ($\bar x = 34.96$, SE = 0.05).

### Monthly and daily trends

**Figure 2:**
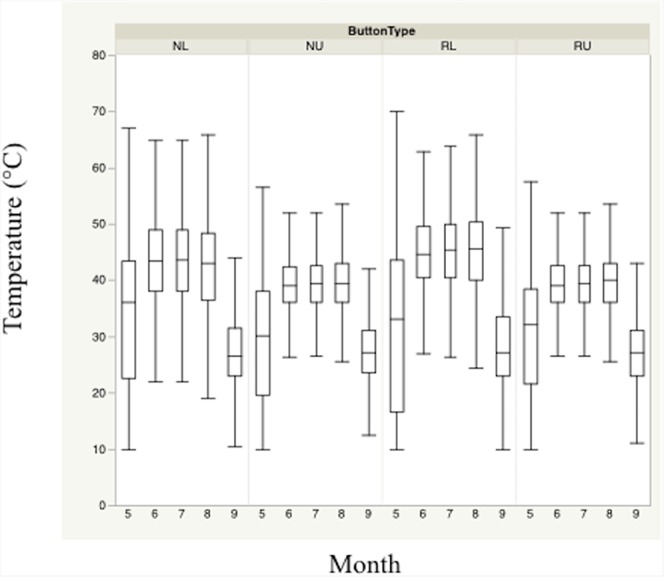
Monthly trends of mean temperature at four sensor location/height combinations. Monthly trends in mean temperature in bunch grasses suitable for bobwhite nesting cover and paired random points by sensor height (∼10 and ∼60 cm) during bobwhite nesting season (May–September) in the rolling plains of Texas, USA, 2012–2014.

**Figure 3:**
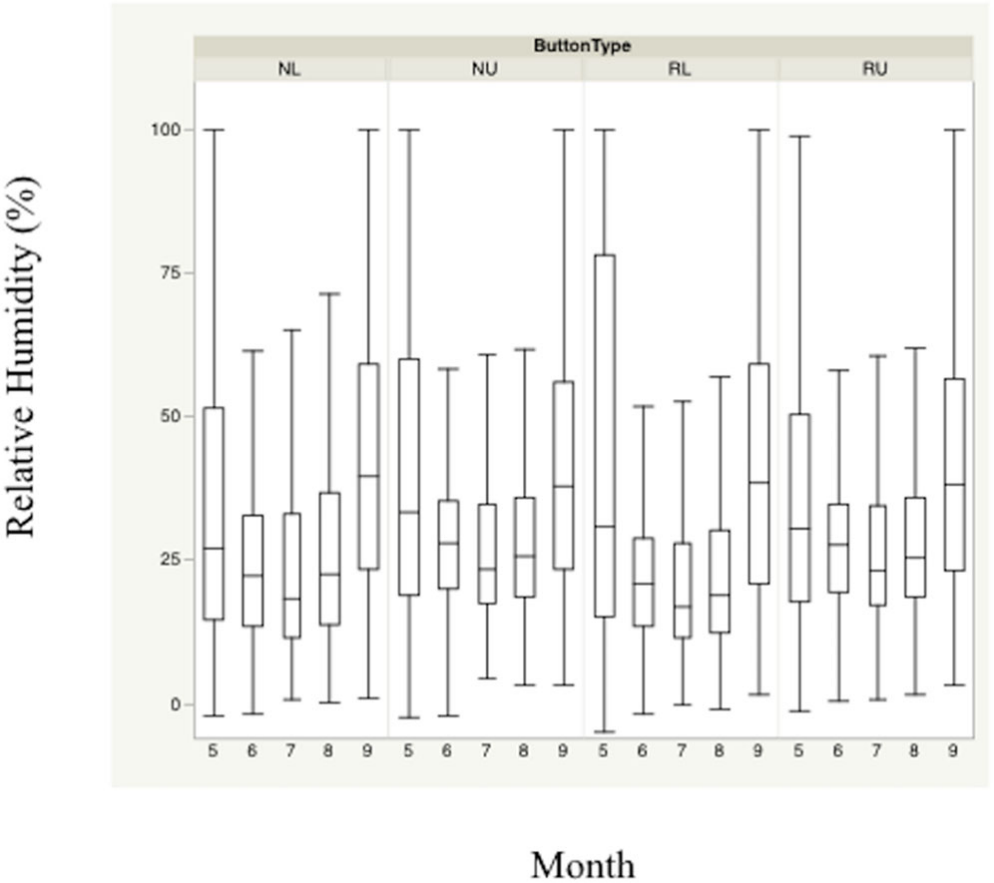
Monthly trends of mean percent relative humidity at four sensor location/height combinations. Monthly trends in mean percent relative humidity in bunch grasses suitable for bobwhite nesting cover and paired random points by sensor height (∼10 and ∼60 cm) during bobwhite nesting season (May–September) in the rolling plains of Texas, USA, 2012–2014.

Monthly means plots indicate divergent mean temperature and relative humidity across months during the nesting season based primarily on height (Figs. 2 and 3). Trends for both temperature and relative humidity were similar, regardless of sensor height or location, although the magnitude of the differences was based primarily on height. Ambient heights exhibited similar trends, and experienced coolest mean temperatures and lowest mean relative humidity throughout the day. Differences in temperatures and relative humidity between height/location combinations were smallest at the beginning and end of the nesting season.

Nest height in nesting locations experienced cooler temperatures and higher relative humidity than nest height in random locations during daily heat maxima. Nevertheless, both nesting and random locations at nest height appeared to maintain harmful and/or lethal temperatures throughout much of the nesting season during daily heat maxima whether viewed by month (Figs. 2 and 3). Cumulatively, 257 days experienced temperatures ≥40 °C at one of our sensors during the 304 sampling days.

### Habitat influences

**Figure 4:**
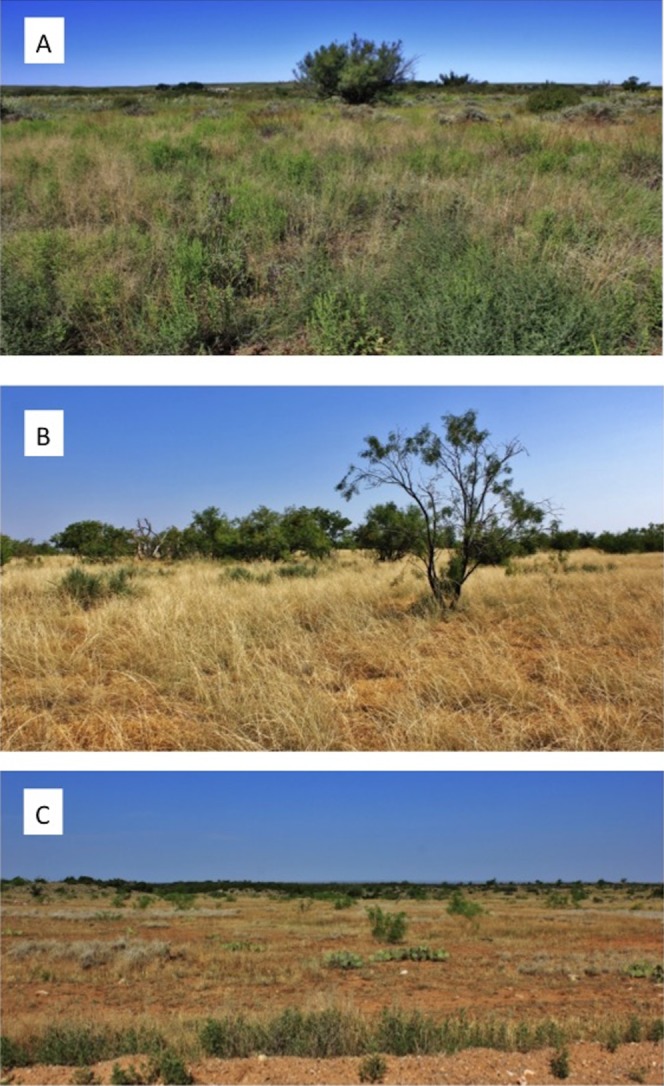
Photographs of range sites delineating (A) excellent, (B) moderate, and (C) poor bobwhite habitat conditions. Photographs that give representative depictions of range conditions that we classified as (A) excellent, (B) moderate, or (C) poor bobwhite habitat for purposes of stratifying data for post-hoc analyses based on relative habitat condition.

**Figure 5:**
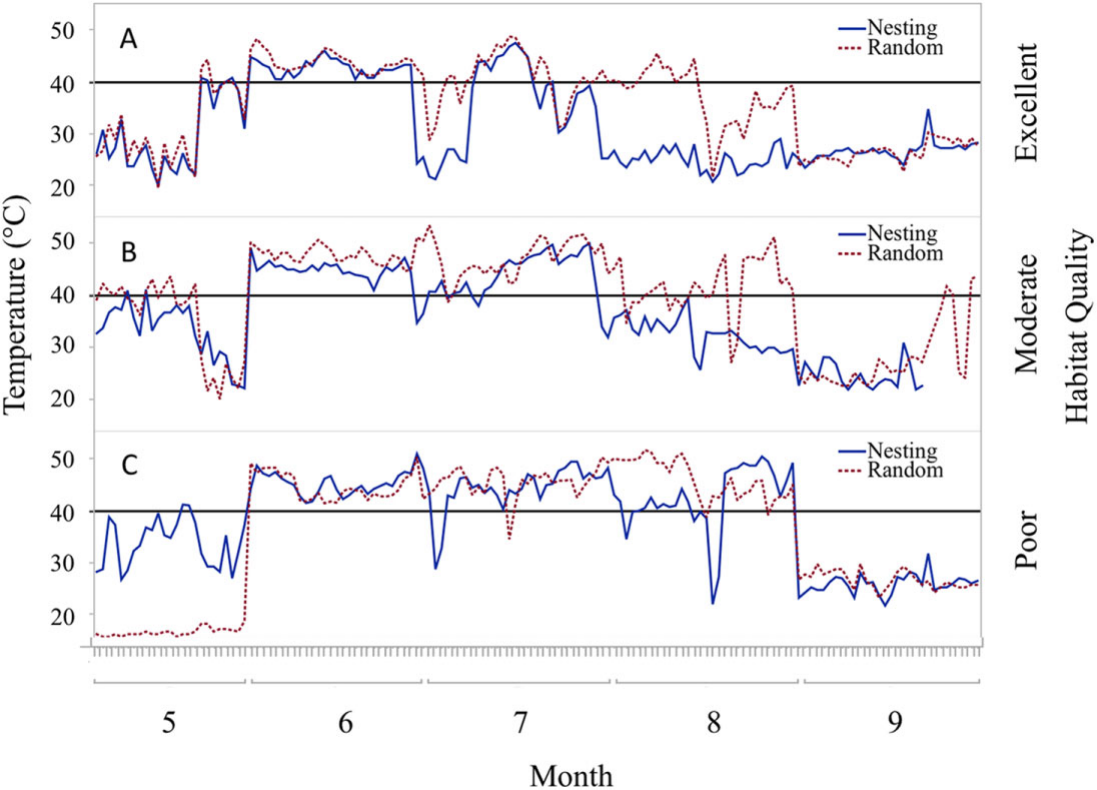
Daily trends of mean temperature at two sensor locations at nest height on three ranches representing excellent, moderate, and poor bobwhite habitat conditions. Daily trends in mean temperature in bunch grasses suitable for bobwhite nesting cover and paired random points by sensor height (∼10 and ∼60 cm) at daily heat maxima during bobwhite nesting season (May–September) on three ranches representing (A) excellent, (B) moderate, and (C) poor habitat conditions in the rolling plains of Texas, USA, 2012–2014. Harmful heat threshold (Reyna & Burggren, 2012) indicated by line at 40 °C.

**Figure 6:**
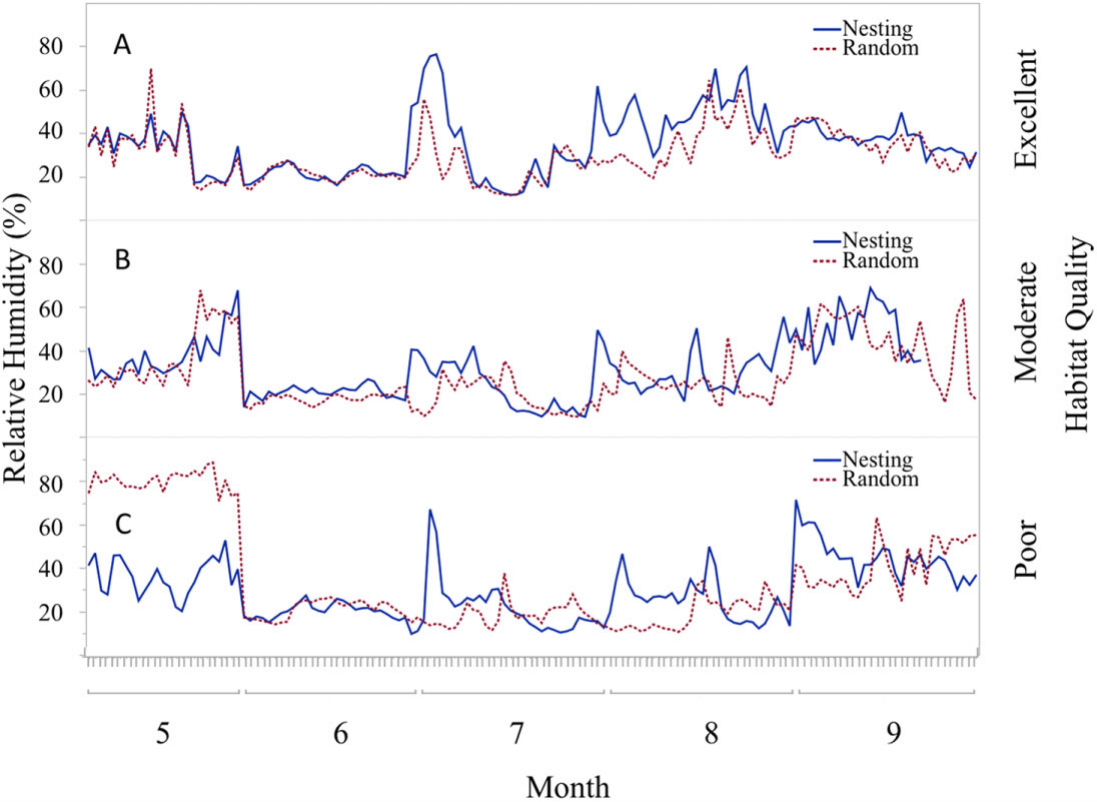
Daily trends of mean percent relative humidity at two sensor locations at nest height on three ranches representing excellent, moderate, and poor bobwhite habitat conditions. Daily trends in mean percent relative humidity in bunch grasses suitable for bobwhite nesting cover and paired random points by sensor height (∼10 and ∼60 cm) at daily heat maxima during bobwhite nesting season (May–September) on three ranches representing (A) excellent, (B) moderate, and (C) poor habitat conditions in the rolling plains of Texas, USA, 2012–2014.

Trends in temperature and relative humidity at nest height in nesting and random locations when viewed by habitat condition (Fig. 4) revealed that temperatures were below harmful levels (∼40 °C) longer in nesting cover than at sites with relatively better habitat conditions (Figs. 5 and 6). Student’s *t-*test revealed that temperature at all habitat classifications were significantly different from one another (*p <* 0.0001). Similarly, a Student’s *t*-test revealed that relative humidity was significantly different from one another at all habitat classifications at a *p* < 0.0001 level.

## Discussion

We found that bunchgrasses can provide thermal refuge for bobwhite nests in the rolling plains on the southwestern periphery of the species range by maintaining a cooler, moister microclimate than surrounding random points. In situ temperature and relative humidity differed significantly primarily as a result of cover type, and to a lesser extent height, during our study. Cooler microclimates in nesting cover result from the insulatory capacity of bunch grasses to resist temperature fluctuation vis-à-vis thermal mass and evaporative cooling, thereby moderating grass temperature at ground height, as well as the air space above it (Barbour, Burk & Pitts, 1999). Some of the temperatures recorded during our study not only meet, but also exceed the thermal thresholds for bobwhites and their eggs documented in laboratory studies (Dabbert, Lochmiller & Teeter, 1997; Reyna, 2010; Reyna & Burggren, 2012). Thus, we conclude that thermal refuges are necessary for bobwhites in semi-arid landscapes, particularly for eggs prior to incubation and adults during incubation, to avoid heat stress that characterizes the semi-arid portion of their range, as suggested by Forrester et al. (1998), Guthery, Land & Hall (2001), and Guthery et al. (2005).

Given that bobwhite eggs would experience potentially lethal heat stress without nesting cover suitable for a thermal refuge, it is logical that such vegetation is a critical component of bobwhite niche space in the semi-arid portion of the species’ range. Because bobwhites reproduce during summer, exhibit limited-dispersal ability, and are a relatively *r*-selected species (Hernández & Peterson, 2007; Lohr et al., 2011; Roseberry & Klimstra, 1984), it is critical that nesting cover suitable for thermal refuges be present across broad spatial extents and through time to maximize bobwhite production and landscape inhabitability (Guthery, 1999).

Ultimately, variability in weather and habitat condition defines the thermal suitability of a landscape for bobwhites. Previous studies demonstrated the strong influence of weather (i.e., precipitation and temperature) on bobwhite abundance in the semi-arid portions of the bobwhite’s range (Bridges et al., 2001; Guthery et al., 2002; Lehmann, 1984; Lusk et al., 2002). Fluctuations in precipitation, including frequent, extended droughts, limits the production and quality of nesting cover in this physiographic region (Lehmann, 1946; Lusk et al., 2006; Sands et al., 2012). Thus, any land use that further reduces the availability of thermal refuge during the nesting season (e.g., excessive livestock grazing, broad scale tillage) would also reduce bobwhite landscape carrying capacity.

During the course of this study, differences in habitat condition within and across ranches due to differences in land use, precipitation, and other factors were apparent. Trends in temperature and relative humidity at nest height in nesting and random locations on three ranches that represent excellent, moderate, and poor habitat conditions, however, revealed that temperatures were below harmful levels (∼40 °C) for longer in nesting cover on ranches with better habitat conditions (Figs. 5 and 6). This situation would increase habitability through time, as well as space, by preserving thermal refuge. Thus, we suggest localized ground temperatures are inversely proportional to vegetative cover, and that reduced solar insolation on bare ground around nesting cover is largely responsible for this refuge effect.

Whether the result of livestock grazing practices, the historic drought affecting the rolling plains during our study, or both, some sites provided nesting cover that would have been thermally suitable for laying, incubation, and hatching quail for a longer portion of the reproductive season than did others. Although bobwhites existed historically under diverse agricultural land uses across their range (small farms to large ranches; Jackson, 1969; Leopold, 1931), the extensification (Morgan-Davies et al., 2014) and/or intensification of land use threatens bobwhites. For example, over the last several decades, traditional agricultural systems have been replaced in the rolling plains by extensive “clean farming” cultivation and excessive cattle grazing (Peterson, Wu & Rho, 2002; USDA-NASS, 2007; Wilkins et al., 2009). These land uses often degrade or destroy bobwhite nesting cover, thereby lowering bobwhite carrying capacity by severely constraining habitat availability through space and time, particularly thermally suitable nesting cover (Guthery et al., 2005).

Where bobwhites persist in semi-arid ecosystems, land uses that maximize agricultural production often produce landscapes that exist at the fringe of the temperature dimension of bobwhite niche space. This portion of niche space expands and contracts with weather variability, but is further constrained by anthropogenic landscape changes that increase temperatures by exposing more bare ground or degrading existing nesting cover (Favreau et al., 2009; Foley et al., 2005). Although such “on-the-knife’s-edge” land management may not appear to destroy bobwhite habitat during normal precipitation years, weather extremes (i.e., extended drought, abnormally high temperature) may raise in situ temperatures in bobwhite nesting cover above threshold lethal limits (Brown, 1978), such as during the drought conditions experienced throughout this study. Thus, areas that might otherwise fulfill habitat requirements are removed from the realized niche of the bobwhite by brinkmanship management (sensu MacNab, 1985). Quail biologists recognize resulting habitat loss, fragmentation, and degradation as key contributors to long-term declines in bobwhite abundance and distribution (Williams et al., 2004). As landscape fragmentation increases, these same factors likely disrupt metapopulation dynamics, and induce localized extinction events (Bascompte & Sole, 1996). In order to decelerate or reverse declining bobwhite abundance and distribution in semi-arid ecosystems, land use practices should be modified to provide all aspects of bobwhite niche space in perpetuity (i.e., continual through both space and time) (Guthery, 1999). These land use practices must be coordinated across broad expanses, maintain adequate thermal refuge throughout the year, and operate reflexively to changing weather conditions (Peterson, 2007).

The influence of thermal stress on survival and reproduction requires species to seek thermal refuge from severe weather conditions, whether bobwhites, other birds (Goldstein, 1984; Salzman, 1982; Thomas, 1984), deer (Parker & Gillingham, 1990), rats (Hendersen & Graham, 1979), lizards (Monasterio et al., 2009; Sinervo et al., 2010), or fishes (McDaniel, Snyder & Connor, 1991). As anthropogenic land use changes continue to accelerate, it is likely that habitat fragmentation will increase, thereby reducing available thermal refuge and effectively constraining realized niche space for a number of species. Thus, changes in land use that exceed thermal limitations to niche space may play an important role in the recent broad scale decline in many species’ abundance and distribution.

The interaction of weather and habitat fragmentation may affect the availability and quality of thermal refuge to mitigate heat stress by increasing temperatures in situ (Delattre et al., 2013; Opdam, 1991). For species whose distribution is characterized by potentially harmful weather (e.g., extreme heat or cold, drought or flood), the loss of such refuge may disrupt metapopulation dynamics at various scales (Monasterio et al., 2009; Vitousek et al., 1997), thereby increasing the chance extinction events (Mantyka-pringle, Martin & Rhodes, 2012). Therefore, it is critical to begin modifying land use practices to minimize their impact on thermal aspects of species’ realized niche space. In order to decelerate or reverse this trend, land use practices must be developed (or redeveloped) (Bignal & McCracken, 1996, 2000; Webb, 1998) that maximize thermal refuge throughout the year. Such practices must identify critical habitat requirements in both space and time to maximize conservation potential for species that require thermal refuge. This practice will become especially critical, given projections of changes in mean temperatures in these regions over the next 100 years; to preserve semi-arid, grassland species, conservationists must enable habitat features to provide the maximum thermal refuge within their ability to do so.

## Supplemental Information

10.7717/peerj.3709/supp-1 Supplemental Information 1 Table S1. Results of two repeated measures analysis of variance (ANOVA) addressing the effects of sensor height (∼10 cm and ∼60 cm), sensor location (bunch grasses suitable for bobwhite nesting cover and paired random points), time (month of the nesting season), and interactions among these factors on temperature (°C) in the Rolling Plains of Texas, USA, 2012–2014.

10.7717/peerj.3709/supp-2 Supplemental Information 2 Table S2. Results of two repeated measures analysis of variance (ANOVA) addressing the effects of sensor height (∼10 cm and ∼60 cm), sensor location (bunch grasses suitable for bobwhite nesting cover and paired random points), time (month of the nesting season), and interactions among these factors on percent relative humidity in the Rolling Plains of Texas, USA, 2012–2014.

## Additional Information and Declarations

### Data Availability

The following information was supplied regarding data availability: Tomecek, John (2017): Inadequate thermal refugia constrains landscape habitability for a grassland bird species. figshare. https://doi.org/10.6084/m9.figshare.1200613.v1
