# Supplementary material for: Inadequate thermal refuge constrains landscape habitability for a grassland bird species
Source: PeerJ. 2017 Aug 18;5:e3709. doi: 10.7717/peerj.3709 (PMC5564388; doi:10.7717/peerj.3709)
Supplement: Supplemental Information 1 — Results of two repeated measures analysis of variance (ANOVA) addressing the effects of sensor height (∼10 cm and ∼60 cm), sensor location (bunch grasses suitable for bobwhite nesting cover and paired random points), time (month of the nesting season), and interactions among these factors on temperature (°C) in the Rolling Plains of Texas, USA, 2012–2014. [file peerj-05-3709-s001.docx]

| Table S1. Results of two repeated measures analysis of variance (ANOVA) addressing the effects of sensor height (~10 cm and ~60 cm), sensor location (bunch grasses suitable for bobwhite nesting cover and paired random points), time (month of the nesting season), and interactions among these factors on temperature (°C) in the Rolling Plains of Texas, USA, 2012–2014. | | | |
| --- | --- | --- | --- |
|  |  | **Repeated Measure** | |
|  |  | Month | |
| **Factor** | | F Ratio | *p* value |
| **Between**  **subject**  **factors** | Location | 165.87 (0.01, 28817) | <0.0001 |
|  | Height | 8.69 (0.0, 28817) | <0.001 |
|  | Location x Height  Interaction | 925.31 (0.03, 28817) | <0.0001 |
| **Within**  **subject factors** | Month | 28468.31 (0.62, 70899) | <0.0001 |
| **Interaction**  **with**  **time** | Location | 19.16 (0.62, 70899) | <0.0001 |
|  | Height | 100.35 (0.62, 70899) | <0.0001 |
|  | Location x Height | 299.60 (0.62, 70899) | <0.0001 |
