# Supplementary material for: Inadequate thermal refuge constrains landscape habitability for a grassland bird species
Source: PeerJ. 2017 Aug 18;5:e3709. doi: 10.7717/peerj.3709 (PMC5564388; doi:10.7717/peerj.3709)
Supplement: Supplemental Information 2 — Results of two repeated measures analysis of variance (ANOVA) addressing the effects of sensor height (∼10 cm and ∼60 cm), sensor location (bunch grasses suitable for bobwhite nesting cover and paired random points), time (month of the nesting season), and interactions among these factors on percent relative humidity in the Rolling Plains of Texas, USA, 2012–2014. [file peerj-05-3709-s002.docx]

| Table S2. Results of two repeated measures analysis of variance (ANOVA) addressing the effects of sensor height (~10 cm and ~60 cm), sensor location (bunch grasses suitable for bobwhite nesting cover and paired random points), time (month of the nesting season), and interactions among these factors on percent relative humidity in the Rolling Plains of Texas, USA, 2012–2014. | | | |
| --- | --- | --- | --- |
|  |  | **Repeated Measure** | |
|  |  | Month | |
| **Factor** | | F Ratio | *p* value |
| **Between**  **subjects**  **factor** | Location | 0.43  (0.00, 28817) | 0.5135 |
|  | Height | 20.678  (0.00, 28817) | <0.0001 |
|  | Location x Height Interaction | 629.01  (0.02, 28817) | <0.0001 |
| **Within**  **subjects factor** | Month | 10428.09  (0.83, 95893) | <0.0001 |
| **Interaction**  **with**  **time** | Location | 34.33  (0.83, 95893) | <0.0001 |
|  | Height | 27.02  (0.83, 95893) | <0.0001 |
|  | Location x Height | 142.91  (0.83, 95893) | <0.0001 |
